# Supplementary material for: Functional ultrasound imaging and neuronal activity: How accurate is the spatiotemporal match?
Source: Imaging Neurosci (Camb). 2025 Jun 16;3:IMAG.a.34. doi: 10.1162/IMAG.a.34 (PMC12319918; doi:10.1162/IMAG.a.34)
Supplement: Supplementary Material [file imag.a.34_supp.pdf]

Supplementary Materials

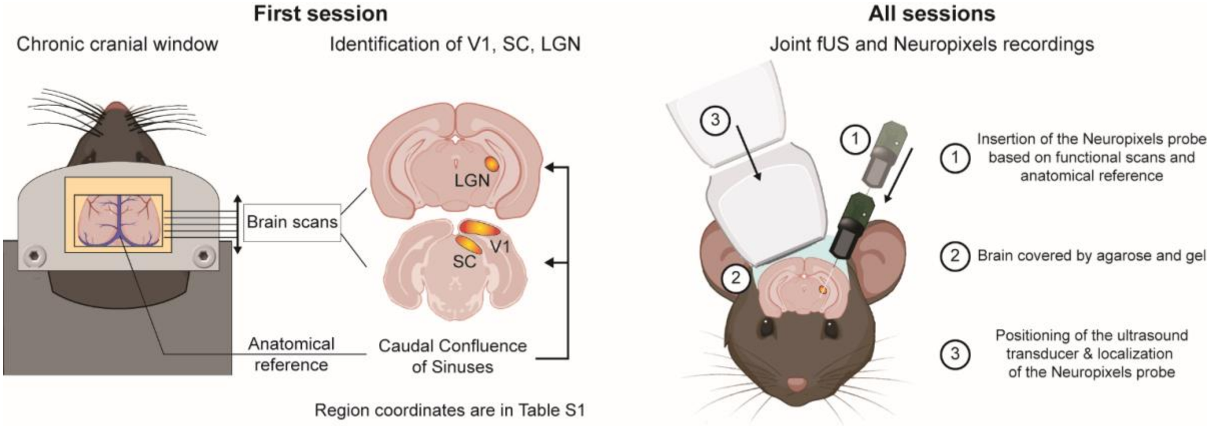

Figure S1. Schematic representation of the data acquisition procedure.

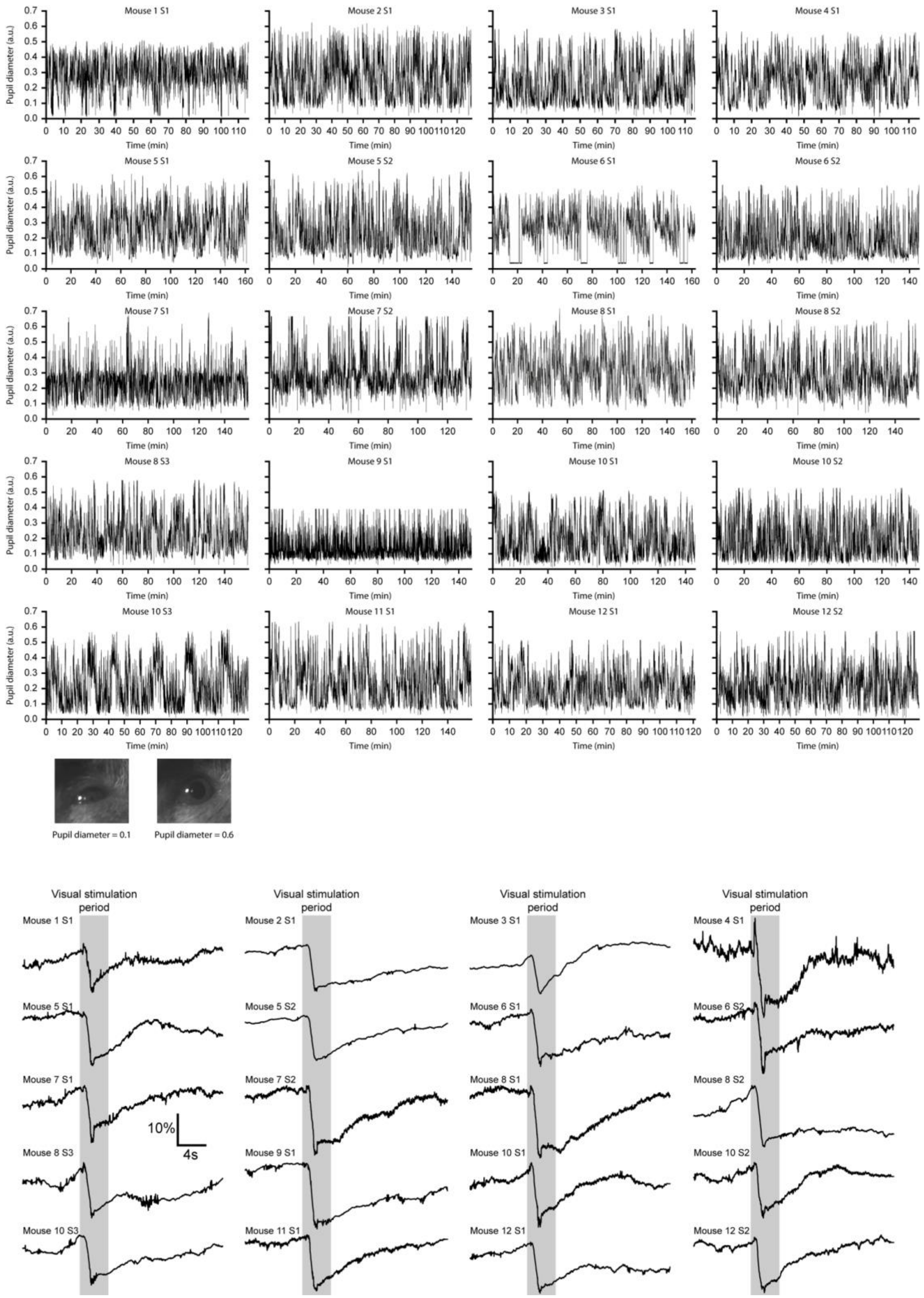

Figure S2. Pupil diameter variation across experimental sessions.
Top, Normalized variation of pupil diameter tracked across sessions confirming for alertness of mice along recordings and example video frames used to compute the mouse's pupil diameter with small (left) and large pupil size (right) with corresponding pupil diameter (a.u.). *Bottom,* Normalize and average temporal traces of pupil dynamics (% of diameter change as compared to baseline) for 50% and 100% contrast conditions for each individual imaging sessions.

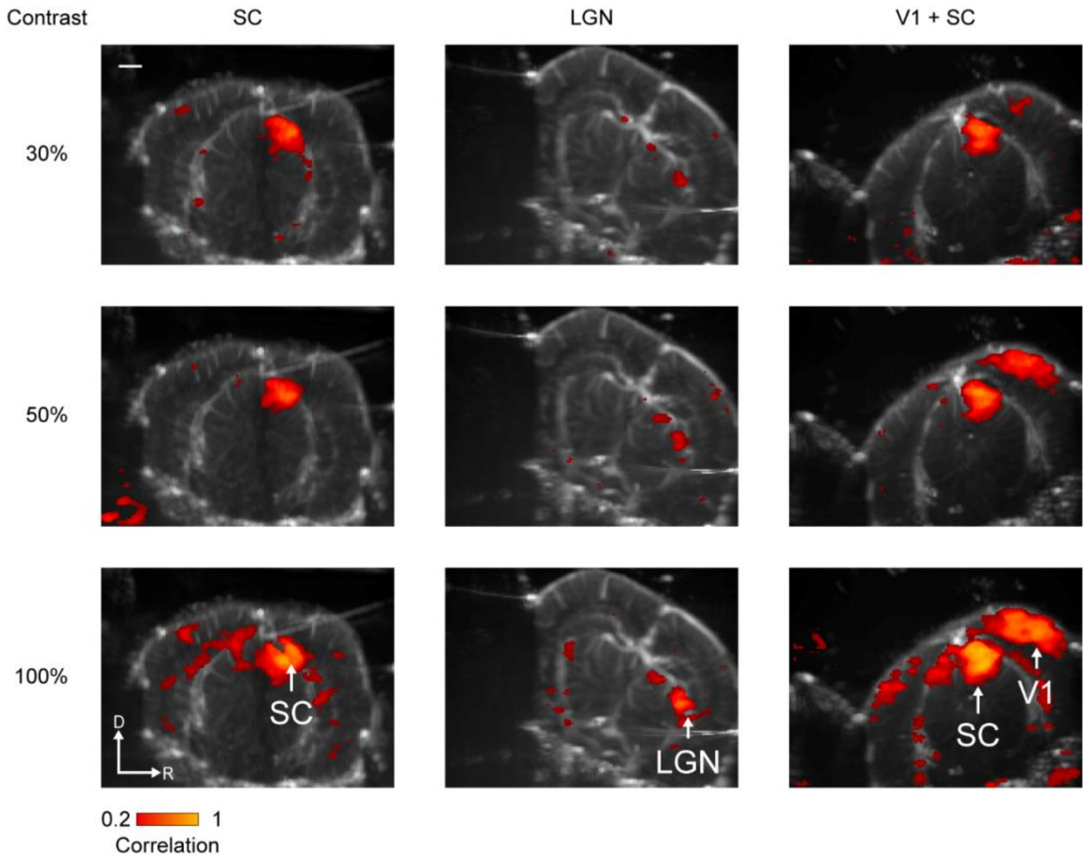

Figure S3. Correlation maps across regions and sessions.
Example of correlation maps generated by computing the correlation between fUS voxels and the stimulation pattern across contrast conditions (50 trials each) and regions of interest (one session per region). Scale bar: 1 mm. D, dorsal; R, right.

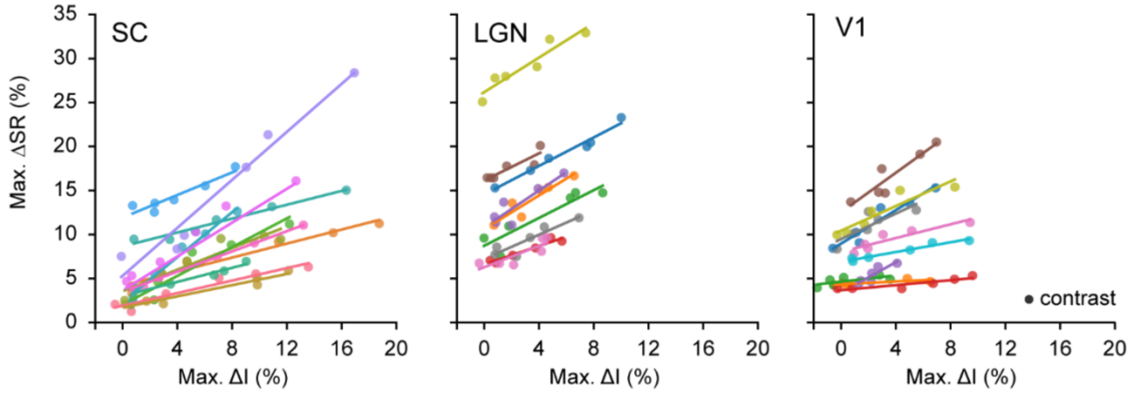

Figure S4. fUS and spike rate activities in responses to contrast conditions. Non-normalized maximum of spike rate variation (max. ΔSR in %) with respect to the maximum of fUS signal variation (max. ΔI in %). Color corresponds to a recording session and dot represents a contrast condition. Basal activity (mean ± sd) for SC: 4.50 ± 3.11, V1: 6.79 ± 3.13, LGN: 10.36 ± 6.46. Proportionality coefficient (mean ± sd) for SC: 0.74 ± 0.46, LGN: 1.06 ± 0.46, V1: 0.55 ± 0.34.

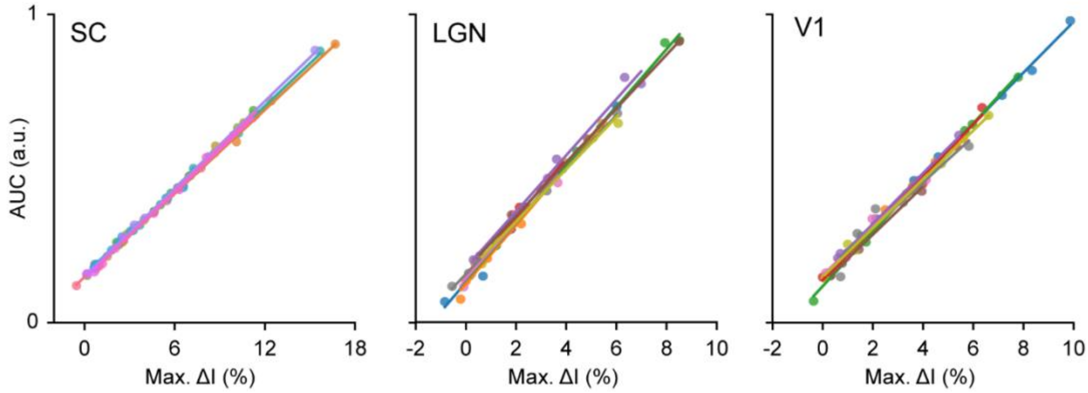

Figure S5. Correlation between maximum ΔI and area under the curve.
Maximum of fUS signal variation (max. ΔI in %) with respect to the area under the curve of fUS signal variation (AUC in arbitrary units, a.u.). Color corresponds to a recording session and dot represents a contrast condition.

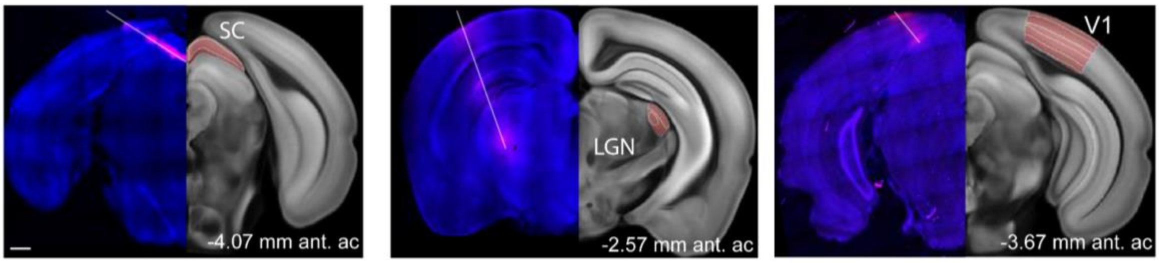

Figure S6. Neuropixels probe trajectories through the mouse brain.
Histological reconstruction of the Neuropixels probe trajectories (white lines) showing DAPI staining (blue) and the fluorescent indicator DiI (pink) used to coat the probe. Trajectories are in the superior colliculus, lateral geniculate nucleus, or primary visual area; from left to right. Scale bar: 0.6 mm.

Table S1. Mice and sessions.

Mouse ID	Number of sessions per region		
	**SC**	**LGN**	**V1**
1	2		
2	1		
3	2		
4	1		
5	1	2	
6	1	1	1
7	1	1	2
8	3	1	3
9		1	
10		2	1
11		1	
12			2
**Total**	12	9	9

Table S2. Estimated parameters of the gamma transfer function per region.

Region	Alpha	Scale
SC	2.9893	2.0645
LGN	1.9305	3.6645
V1	3.5801	2.2239

Table S3. Coordinates used for Neuropixels probe insertion. CCS: Caudal confluence of Sinuses.

Regions of interest	Position relative to CCS (mm)	Lateral position from midline (mm)	Angle (relative to horizontal)	Insertion depth (mm)
**SC**	+0.5	2.5	25°	3.0
**V1**	+0.4	3.0	12°	3.5
**LGN**	+1.6	3.6	60°	3.5
